# A novel single-chain antibody redirects adenovirus to IL13Rα2-expressing brain tumors

**DOI:** 10.1038/srep18133

**Published:** 2015-12-14

**Authors:** Julius W. Kim, Jacob S. Young, Elena Solomaha, Deepak Kanojia, Maciej S. Lesniak, Irina V. Balyasnikova

**Affiliations:** 1 The Brain Tumor Center, The University of Chicago, Chicago, IL 60637, USA; 2 Biophysics Core Facility, The University of Chicago, Chicago, IL 60637, USA

## Abstract

The generation of a targeting agent that strictly binds to IL13Rα2 will significantly expand the therapeutic potential for the treatment of IL13Rα2-expressing cancers. In order to fulfill this goal, we generated a single-chain antibody (scFv47) from our parental IL13Rα2 monoclonal antibody and tested its binding properties. Furthermore, to demonstrate the potential therapeutic applicability of scFv47, we engineered an adenovirus by incorporating scFv47 as the targeting moiety in the viral fiber and characterized its properties *in vitro* and *in vivo.* The scFv47 binds to human recombinant IL13Rα2, but not to IL13Rα1 with a high affinity of 0.9 · 10^−9^ M, similar to that of the parental antibody. Moreover, the scFv47 successfully redirects adenovirus to IL13Rα2 expressing glioma cells both *in vitro* and *in vivo*. Our data validate scFv47 as a highly selective IL13Rα2 targeting agent and justify further development of scFv47-modified oncolytic adenovirus and other therapeutics for the treatment of IL13Rα2-expressing glioma and other malignancies.

Glioblastoma (GBM) is the most common and deadly malignant brain cancer in adults with limited therapeutic options^1^. The current therapeutic approaches do not discriminate neoplastic cells from normal cells resulting in undesirable toxicity. In that respect, new targeted approaches for GBM treatment are in urgent need. Specifically with regard to virotherapy for GBM, a lack of specific targeting is usually balanced by conditional replication of oncolytic adenovirus in cancer cells^2^,^3^,^4^,^5^. For example, the conditionally-replicative (CRAd) therapeutic virus (CRAd-S-pk7) has lysine residues added to the fiber knob domain to enhance infectivity, while the viral replication is simultaneously restricted to glioma cells by controlling the replication of the essential early gene (E1) expression under the tumor-specific survivin promotor^5^. This therapeutic agent has demonstrated efficacy in preclinical models^5^,^6^. However, the presence of low survivin promoter activity in non-neoplastic cells^4^ and variable activity amongst gliomas^7^,^8^ dictates the development of additional specific targeting strategies.

Given this need, several research efforts have pursued the development of more specific therapeutic agents for GBM by targeting tumor-specific cell surface proteins^9^,^10^,^11^,^12^,^13^. One tumor-specific target is IL13Rα2, an IL13 receptor variant that is selectively expressed on glioma cells^14^,^15^,^16^. Furthermore, IL13Rα2 expression in glioblastoma has been associated with an increased malignancy grade, the aggressive mesenchymal gene expression signature, and higher IL13Rα2 expression has been correlated with a poorer patient prognosis^17^, all of which suggest that treatment approaches that can specifically target IL13Rα2-expressing glioma cells could improve outcomes for patients with a particularly aggressive tumor phenotype. As such, the selective targeting of this glioma-specific protein is a highly promising strategy for oncolytic, cytotoxic, and immunotherapeutic agents that depend on a high level of specificity for efficacy and safety.

The use of IL13 ligand itself is one way to direct therapy to the glioma-specific IL13Rα2. This approach has been used to redirect adenoviral tropism and a mutated form of the IL13 ligand has been used to direct CAR T-cells (e.g. IL13-zetakine T cells)^10^,^11^ or adenovirus^18^ to this tumor specific receptor. However, even the modified IL13 ligand still interacts with the IL13Rα1^10^,^11^, another receptor for IL13, which is widely expressed by normal cells, underlining the unmet need for exclusive IL13Rα2 targeting agents.

To address this problem, an IL13Rα2-specific monoclonal antibody (mAb) has been developed and has been shown to bind to the IL13Rα2 with exclusive specificity. This antibody also competes with IL13 for its binding site on IL13Rα2 and has been shown to improve survival in mice with an intracranial model of glioblastoma^19^, as well as mice with colorectal cancer^20^. However, the therapeutic applicability of the whole mAb is limited by its rather large size, making it difficult to incorporate into other therapeutic reagents, as well as by its murine origin, which renders it capable of generating undesirable immune responses. For this reason, we engineered a single-chain variable antibody fragment (scFv47) derived from our well characterized IL13Rα2-specific monoclonal antibody^19^.

In the study, we demonstrate that our novel scFv47 possesses exclusive specificity of binding to IL13Rα2 and recognizes the same epitope as the parental monoclonal antibody with a high affinity. Furthermore, as a proof of principle to show its potential therapeutic applicability, we generated a novel IL13Rα2-targeted adenoviral vector by incorporating this scFv47 into the fiber knob domain of a fiber-fibritin modified adenovirus^21^,^22^,^23^. We demonstrate that this fiber modification successfully redirects the viral tropism specifically to IL13Rα2-expressing glioma cells *in vitro* and *in vivo*. Our data validate that scFv47 represents a valuable reagent necessary for development of personalized therapeutics that are tailored specifically to a patient’s tumor phenotype.

## Results

### Cloning and characterization scFv fragment of mAb47

**Figure 1:**
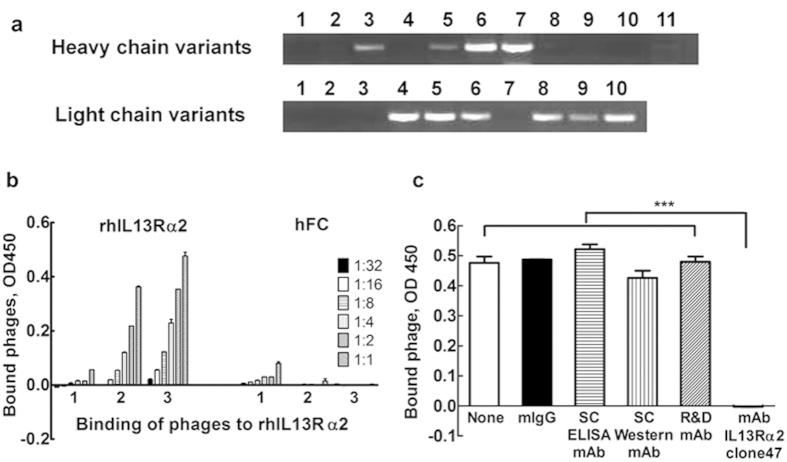
Generation and characterization of scFv47. (**a**) Screening of parental hybridoma IL13Rα2 cells mRNA for Vh and Vl using set Vh and Vl specific primers (Suppl. Table 1). (**b**) ELISA testing for phage binding enriched in three rounds of biopanning against rhIL13Rα2 protein and hFc as a negative control. (**c**) Competitive Assay. Plates coated with rhIL13Rα2 protein were treated with a panel of anti-IL13Rα2 mAbs that recognize non-overlapping epitopes with the parental mAb IL13Rα2 (clone 47)^19^. Binding of phages in the absence or presence of antibodies was analyzed via detection with anti-M13 phage antibody as described in the Material and Methods section. Each data point is an average of 3 independent replicates in all figures. Data presented as mean ± SEM. ***p < 0.001.

We previously reported that our new mAb IL13Rα2 (clone 47) possess unique properties, such as a strong binding with the native epitope of IL13Rα2, high affinity of interaction with IL13Rα2, and the ability to compete with IL13 for its recognition site on IL13Rα2^19^. Here, we report the engineering of scFv47 from the hybridoma cell line secreting parental antibody. The analysis of mRNA from this hybridoma cell line revealed the presence of multiple transcripts corresponding to the heavy and light chains of mAb (Fig. 1a)^24^,^25^. In order to obtain a functional combination of heavy chain and light chain variable regions assembled in the scFv47, we utilized a phage–display approach^24^,^25^. For that reason, the library of phages for the heavy and light chain variants in a scFv format was generated and screened against recombinant human IL13Rα2 (rhIL13Rα2) as described below.

After generating scFv47, we verified its binding specificity to rhIL13Rα2. To this end, supernatant containing phages during all rounds of bio-panning were generated and assayed for binding specificity to rhIL13Rα2 protein by ELISA. Figure 1b shows that the specificity phage interaction dramatically increases with each subsequent round of biopanning, whereas non-specific binding to the negative control, human IgG decreases to an undetectable level. After the last round of biopanning, individual phage clones were generated. Sequence analysis of XL1 blue *E. coli* infected with individual phage clones revealed that all selected phages contained an identical sequence of the scFv47. Thus, these data indicate that scFv47 was successfully generated through three rounds of phage biopanning.

Next, we performed a competitive assay to determine if the newly cloned scFv47 and the parental mAbIL13Rα2 (clone 47) bind to the same epitope on the IL13Rα2 molecule. Figure 1c shows that mAb IL13Rα2 (clone 47) completely prevented the interaction of scFv47 expressing phages to immobilized rhIL13Rα2 protein. Neither the control mIgG nor three other mAbs against IL13Rα2, which recognize non-overlapping epitopes of the mAb IL13Rα2(clone 47) on human IL13Rα2^19^, interfered with the binding of scFv47 to IL13Rα2. This result clearly demonstrates that scFv47 shares the same recognition site on IL13Rα2 as parental monoclonal antibody.

**Figure 2:**
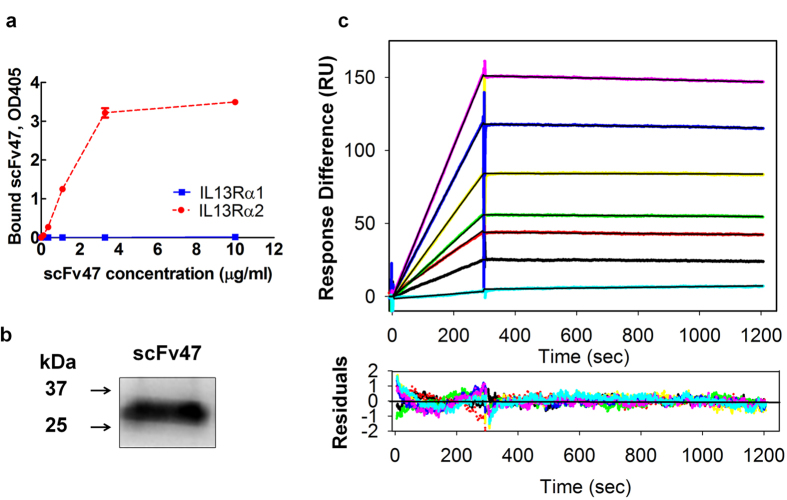
Binding characteristics of scFv47 to IL13Rα2. (**a**) Binding of purified soluble scFv47 with rhIL13Rα2 and rhIL13Ra1 proteins was determined in plate ELISA. (**b**) Western blot analysis of soluble scFv47. The scFv47 protein runs under reducing conditions as a 30kDa protein in agreement with the predicted molecular weight. (**c**) The kinetics of interactions between the scFv47 and rhIL13Rα2 were visualized by SPR in a Biacore 3000. The scFv47 was injected at concentrations ranging from 1 to 50 nM (lower to upper curves) at a constant flow rate of 20 μL/min over immobilized rhIL13Rα2. The association phase was monitored for 30 sec, dissociation phage for 900 sec following by the change in SPR signal (colored curves), given in RU. Black curves represent the fit of the data to a one-site binding model. For derived kinetic parameters, see Table 1. Lower panels show residuals from the one-site binding model, indicating an excellent fit.

The specificity of binding of soluble scFv47 to IL13Rα2 was further validated. For that, soluble scFv47 was generated in XL1 blue *E. Coli* and purified as described in the Materials and Methods. An analysis of the binding of soluble scFv47 in plate ELISA demonstrated lack of interaction with rhIL13Rα1 and specific binding with rhIL13Rα2 (Fig. 2a). Figure 2b shows that the molecular weight of soluble scFv47 protein is about 30 kDa, which corresponds to its predicted value. Furthermore, the plasmon resonance analysis revealed that soluble scFv47 binds to rhIL13Rα2 with a high affinity (0.9 · 10^−9^ M) similar to that of the parental antibody^19^ (Table 1 and Fig. 2c). Thus, the obtained scFv47 was deemed to be fully functional as determined by a specific interaction of scFv47–expressing phages and soluble scFv47 to rhIL13Rα2.

### Generation of IL13Rα2 targeted adenoviral vector

**Figure 3:**
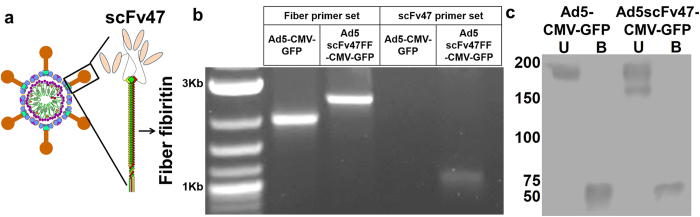
Design, Generation, and Confirmation of IL13Rα2 Tropic Virus Structure and Stability. (**a**) Schematic diagram of anti-IL13Rα2 scFv-specific chimera fiber of Ad5FFscFv47-CMV-GFP. The fiber knob and shaft domains of Ad5 were replaced with a fiber fibritin trimerization domain, and anti-IL13Rα2 scFv47 was incorporated into the C-terminus of the chimeric fiber. (**b**) PCR confirmation of fiber modification. (**c**) Validation of the chimeric fiber structure. Western blot analysis detected the stable fiber trimerization when the chimeric fiber was unboiled (U: incubated at room temperature for 10 min), and detected denatured monomeric structures when the fiber was boiled (B: incubated at 95 °C for 10 min).

In order to redirect the viral tropism to IL13Rα2, we genetically modified both the viral fiber shaft and knob domains^22^,^26^. First, the shaft domain was replaced with a fiber-fibritin (FF) trimerization domain to ensure stability of the binding motif structure, and then the scFv47 was incorporated in the C-terminal of the FF shaft domain (Fig. 3a). The purified Ad5scFv47FF-CMV-GFP virus titer was comparable to that of Ad5-CMV-GFP, indicating that scFv47 incorporation does not affect the yield of virus production (Suppl. Fig. 1). To confirm the genetic modification of the fiber, PCR analysis was performed with purified viral DNAs using either fiber-specific or scFv47-specific primer sets. Figure 3b demonstrates the successful incorporation of scFv47 in the FF domain of adenovirus (Ad5FFscFv47). Furthermore, western blot analysis of the wild-type and recombinant virus further confirmed (Fig. 3c) that the new chimeric fiber has a composition similar to that of the wild-type fiber, indicating that incorporation of scFv47 does not hinder the fiber’s trimerization or cause structural instability of the fiber.

### Demonstration of CAR independent infection

**Figure 4:**
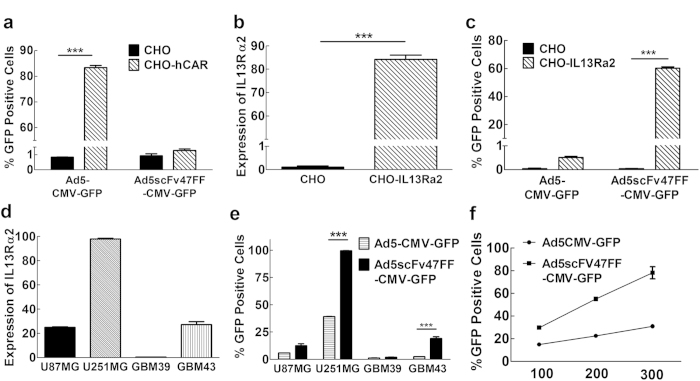
Confirmation of Tropism Modification of Ad5FFscFv47-CMV-GFP. (**a**) CAR-independent infectivity of Ad5FFscFv47-CMV-GFP virus. CAR-negative CHO and CAR-positive CHO-hCAR cell lines were infected with Ad5CMV-GFP or Ad5FFscFv47-CMV-GFP virus. Cells were analyzed for GFP expression 72 hours post infection by flow cytometry. (**b**) The expression of IL13Rα2 on the surface of CHO-IL13Rα2 cell line detected using mAb IL13Rα2 (clone 47). (**c**) IL13Rα2-dependent infectivity of Ad5FFscFv47-CMV-GFP demonstrated by efficient transduction of CHO-IL13Rα2 cells and lack of transduction of IL13Rα2-negative CHO cells. (**d**) The IL13Rα2 expression on the surface of U87MG, U251MG, GBM39, and GBM43 glioma cell lines. Data presented as percent of positive cells. (**e**) The transduction efficiency of Ad5FFscFv47-CMV-GFP, but not Ad5CMV-GFP virus, strongly correlates with a level of IL13Rα2 expression in U87MG, U251MG, GBM39, and GBM43 glioma cells. Transduced glioma cells were analyzed by flow cytometry for GFP expression 72 hours post infection. (**f**) Steady increase in the infectivity of Ad5FFscFv47-CMV-GFP with an increase of MOI. U251MG cells were infected with Ad5FFscFv47-CMV-GFP or Ad5CMV-GFP at MOI: 100, 200, and 300 vp/cell. 72 hours post infection, a flow cytometric analysis for GFP expression in cells was performed. Each data point is an average of 3 independent replicates in all figures. Data presented as mean ± SEM. ***p < 0.001.

The primary receptor for the human adenovirus serotype 5 (Ad5) is the *coxsackie and adenovirus receptor* (CAR)^27^,^28^,^29^. It is anticipated, therefore, that Ad5FFscFv47 virus will infect cells in CAR-independent fashion. To confirm that our modification results in a loss of CAR-binding ability, the viral infectivity of Ad5FFscFv47 encoding for green fluorescent protein under the control of CMV promoter (Ad5FFscFv47-CMV-GFP) and wild-type virus, Ad5-CMV-GFP, was analyzed in the human CAR (hCAR)-negative and hCAR-positive (CHO-hCAR) Chinese hamster ovary cell lines. Figure 4a demonstrates that wild-type Ad5-CMV-GFP efficiently infects the CHO-hCAR, but not the hCAR-negative CHO cells, whereas Ad5FFscFv47-CMV-GFP shows very little infectivity in either cell line, indicating on loss of CAR-binding ability.

### IL13Rα2 specific infection of glioma cells *in vitro*

Next, in order to demonstrate IL13Rα2-specific infectivity by the Ad5FFscFv47-CMV-GFP virus, we used CHO and CHO-IL13Rα2 cell lines^19^. As shown in Fig. 4b,c, Ad5FFscFv47-CMV-GFP efficiently infects CHO-IL13Rα2 cells (80% of the IL13Rα2 cells are GFP positive), but not control, IL13Rα2-negative, CHO cells. For further validation of IL-13Rα2-dependent infectivity of Ad5FFscFv47-CMV-GFP, we performed an analysis of viral transduction in U87MG and U25MG cell lines, as well in patient-derived primary GBM43 and GBM39 glioma cells, which endogenously express IL13Rα2 at different levels (Fig. 4d). The flow cytometry analysis revealed that infectivity of Ad5FFscFv47-CMV-GFP strongly correlated with the level of IL13Rα2 expression in the assayed cell lines. GFP expression was observed in about 20% of U87MG and 90% of U251MG glioma cell lines, as well in 1% and 20% of GBM39 and GBM43 glioma cells, respectively (Fig. 4e). In contrast, wild-type Ad5-CMV-GFP infection of both glioma cell lines was observed at a very low level, in accordance with the well-characterized low level of CAR expression by glioma cells^30^,^31^. Importantly, the infectivity of Ad5FFscFv47-CMV-GFP in U251MG cells increased proportionally with the viral multiplicity of infection (MOI) used in assay (Fig. 4f), without causing any observable cytotoxicity.

**Figure 5:**
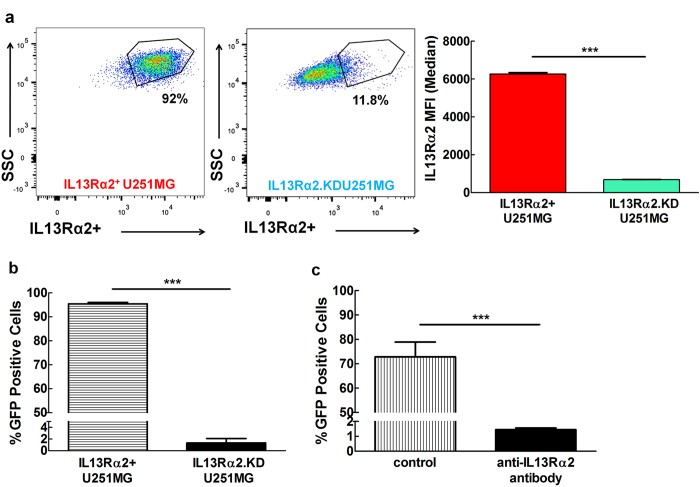
IL13Rα2-specific Infectivity of Ad5FFscFv47-CMV-GFP. (**a**) Flow cytometry analysis of IL13Rα2 expression in U251MG cells following knockdown with control shRNA (IL13Rα2^+^ U251MG) or IL13Rα2-specific shRNA (IL13Rα2KDU251MG) presented as percent of positive cells (flow charts) and median fluorescent intensity (MFI). (**b**) IL13Rα2-dependent infectivity of Ad5FFscFv47 -CMV-GFP demonstrated by differential expression of GFP in in IL13Rα2^+^ U251MG and IL13Rα2.KDU251MG cell lines. (**c**) Competitive binding assay. U251MG cells were pre-treated with anti-IL13Rα2 mAb as described in the Material and Methods section. Control and treated cells were then infected with Ad5scFv47-CMV-GFP virus. Cells were analyzed for GFP transgene expression 72 hours later by flow cytometry. Each data point is an average of 3 independent replicates. Mean ± SEM is plotted. ***p < 0.001.

To further validate IL13Rα2-dependent infectivity of Ad5FFscFv47-CMV-GFP, we generated two cell lines, IL13Rα2^+^ U251MG and IL13Rα2.KDU251MG via transduction with lentivirus encoding either control or IL13Rα2-specific shRNA, respectively. IL13Rα2^+^ U251MG cells retained a very high (above 92% positive cells) expression of IL13Rα2 on their cell surface after lentiviral transduction, whereas IL13Rα2.KDU251MG glioma cells were mostly IL13Rα2 negative (about 12% of IL13Rα2-positive cells) (Fig. 5a). Respectively, Ad5FFscFv47-CMV-GFP infected over 90% of IL13Rα2^+^ U251MG and less than 5% of IL13Rα2.KDU251MG cells (Fig. 5b). Moreover, anti-IL13Rα2 mAb effectively inhibited the infection of U251MG cells by Ad5FFscFv47-CMV-GFP (Fig. 5c) in a competitive assay. Collectively, these data demonstrate that, similarly to the scFv47 protein, the Ad5FFscFv47 is an IL13Rα2-specific viral agent.

### Infection of stem-like cancer glioma cells by Ad5FFscFv47 virus

**Figure 6:**
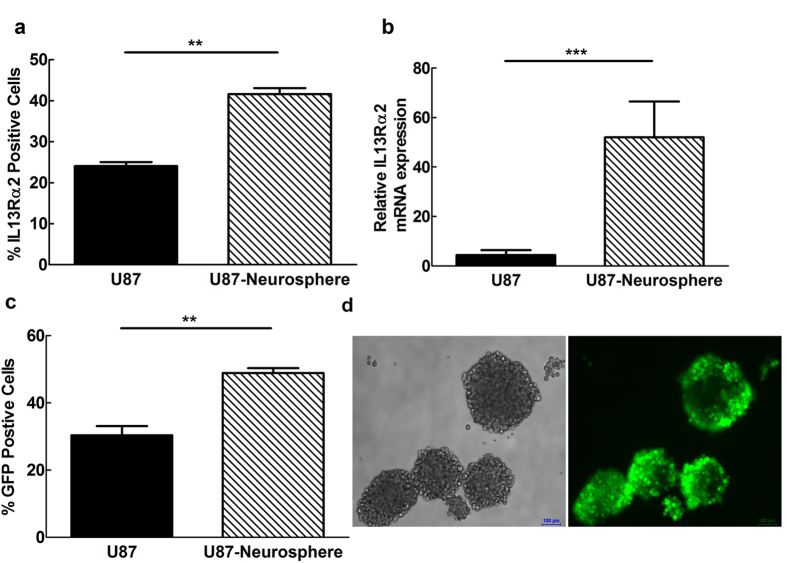
Infection of neurospheres by Ad5FFscFv47-CMV-GFP. (**a**) Comparison of IL13Rα2 expression in U87MG cells growing as an adherent culture or as neurospheres. (**b**) Relative IL13Rα2 mRNA expression in U87MG glioma cells grown as adherent culture or as neurospheres was analyzed by RT-PCR. (**c**) Ad5FFscFv47-CMV-GFP infectivity of U87MG glioma cells grown as adherent culture versus neurospheres was determined by flow cytometry analysis for GFP-positive cells (**d**) Microscopic image of U87MG neurospheres (phase-contrast image-left panel) infected with Ad5FFscFv47-CMV-GFP. GFP expression (right panel) is shown in green fluorescence. Scale bar is 100 μm. Each data point is an average of 3 independent replicates. Mean ± SEM is plotted. ***p < 0.001, **p < 0.01.

As cancer stem cells have emerged as a potential target for glioblastoma treatments, we mimicked cancer stem cell-like properties *in vitro* by culturing the U87MG cells as neurospheres (see the Material and Methods section for details). Analysis of IL13Rα2 expression revealed that U87MG neurospheres had 13 times higher expression of mRNA and 1.7 times higher surface protein expression, respectively, in comparison to cells grown in attached (e.g. differentiated) form (Fig. 6a,b). Accordingly, Ad5FFscFv47-CMV-GFP infectivity was about 1.6 times higher in neurospheres than in adherently growing U87MG cells (Fig. 6c,d). To further validate the ability of Ad5FFscFv47-CMV-GFP to transduce neurospheres derived from the of IL13Rα2-expressing glioma cells, we analyzed patient derived primary glioma cells GBM39 and GBM43. While we observed a slight increase in IL13Rα2 mRNA expression in both GBM39 and GBM43 cell lines cultured as neurospheres, there was no detectable change in the surface expression of IL13Rα2 or the infectivity with Ad5FFscFv47-CMV-GFP in either cell line (Suppl. Fig. 2). The Ad5FFscFv47-CMV-GFP infectivity was well correlated with the level of IL13Rα2 expression on the cell surface in all studied glioma cell lines. Thus, our data suggest that targeting of IL13Rα2 overexpressing cancer stem cells is highly feasible via utilization of scFv47-targeted therapeutic agents such as scFv47-engineered adenovirus.

### Demonstration of IL13Rα2-specific infection *in vivo*

**Figure 7:**
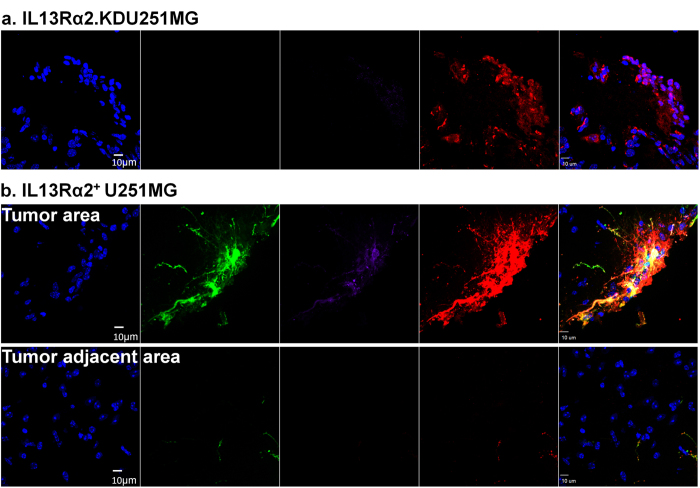
IL13Rα2-specific infection in xenograft model of glioma. Mouse brains were sectioned and stained for DAPI (Blue), GFP (viral infection), anti-GFP (Purple), and anti-human nestin (Red, tumor). (**a**) Immunohistochemistry analysis of IL13Rα2.KDU251MG cells implanted mice. There were no observable GFP-positive cells in tumor area. (**b**) Immunohistochemistry analysis of IL13Rα2^+^ U251MG cell implanted mice. While GFP positive cells were observed in tumor area, there was not observable virus infected cells in the tumor adjacent area, indicating the infectivity of Ad5FFscFv47-CMV-GFP is highly specific to the IL13Rα2 expression level. Scale bar is 10 μm.

Collectively, the *in vitro* assays demonstrate that Ad5FFscFv47-CMV-GFP specifically infects IL13Rα2-expressing glioma cells. Next, we validated the specificity of Ad5FFscFv47-CMV-GFP *in vivo* using intracranially implanted IL13Rα2^+^ U251MG and IL13Rα2.KDU251MG cells in a xenograft murine model of glioma. No infection was detected in the mostly IL13Rα2-negative IL13Rα2.KDU251MG xenograft tissue or in the brain tissue surrounding the tumor as judged by the lack of GFP transgene expression (Fig. 7a). However, we observed a high infection of IL13Rα2-expressing IL13Rα2^+^ U251MG xenograft; and once again there was no detectable GFP signal in the brain tissue adjacent to the tumor (Fig. 7b). Thus, our data confirmed that Ad5FFscFv47-CMV-GFP is capable of specific transduction of IL13Rα2-expressing tumor cells not only *in vitro* but also *in vivo*.

## Discussion

Here, we report a novel scFv47 generated from our parental antibody^19^ through a phage–display approach. We show that the scFv47 specifically recognizes native IL13Rα2 with an exclusively high affinity and recognizes the same epitope as a parental mAb IL13Rα2 (clone 47). Furthermore, we show that the scFv47 can be successfully incorporated into an adenoviral vector and that the Ad5FFscFv47-CMV-GFP virus also exclusively infects IL13Rα2-expressing glioma cells *in vitro* and *in vivo*.

A series of immunotherapeutic agents targeting IL13Rα2 have demonstrated preclinical promise^10^,^11^,^32^,^33^, however these agents also recognize the widespread IL13Rα1, indicating that there is unmet need for specific targeting agents^10^,^11^,^34^. In order to improve the specificity of IL13Rα2 targeting, a monoclonal antibody that exclusively recognizes a native epitope on the IL13Rα2 protein has been previously generated in our laboratory^19^. Engineered antibody fragments, however, have an advantage over the whole antibody, because they can be easily genetically manipulated and incorporated in therapeutic agents. With that in mind, we genetically engineered scFv47 as an IL13Rα2-specific targeting moiety for various future therapeutic agents.

Here, we present our initial efforts to demonstrate that scFv47 has therapeutic applicability by redirecting tropism of the commonly used anticancer agent, adenovirus, via modification of its fiber. Although the incorporation of scFv into adenoviral fiber is known to be difficult due to the stability of scFv itself and that of fiber trimerization^21^,^23^, the scFv47-modified fiber demonstrated stability comparable to that of the wild-type adenoviral fiber, and Ad5FFscFv47-CMV-GFP was no longer able to recognize the native adenoviral receptor, CAR. Instead, the virus exclusively infected IL13Rα2-expressing cells both *in vitro* and *in vivo*. Our results validate scFv47 as a highly selective IL13Rα2 targeting agent and confirm that it can be utilized for the redirection of adenoviral tropism to cancer and cancer stem-like glioma cells. It opens venues for the exploration of scFv47-based viral, cellular, protein, and nanoparticle therapeutics for the treatment of various IL13Rα2-expressing human malignancies.

Recently, research has focused on identifying therapeutic agents that can successfully eradicate cancer stem cells, which are resistant to traditional anticancer therapies and thought to be responsible for cancer recurrence following therapeutic treatment^35^. Based on these properties, glioma stem cells are a highly attractive subset of tumor cells for therapeutic targeting. In agreement with previous reports^36^,^37^, we observed that expression of IL13Rα2 is maintained in primary patient-derived glioma cell growing as neurospheres, which permitted efficient transduction of these cells by Ad5FFscFv47-CMV-GFPvirus. IL13Rα2 expression has recently been associated with an increased malignancy grade and a poorer patient prognosis^17^. Thus, providing a treatment option that specifically targets IL13Rα2-expressing stem-like and differentiated glioma cells would be of benefit to the patients with some of the most aggressive and hardest to treat cancers.

Previously, it has been shown that nearly 50% of GBM patients have tumors that express IL13Rα2^38^, a higher percentage than the other commonly used glioma specific marker EGFRvIII^39^, which indicates significance of this molecular target for the majority of GBM patients^38^. Additionally, following glioma cell death, the phenomenon of ‘epitope spreading’ might enhance the immune response against the tumor and result in further removal of the tumor, even those cells that do not express the originally targeted antigen^40^. As more glioma-specific agents are developed, personalized treatment cocktails can be administered in the future to achieve heightened specificity and efficacy for a given patient’s glioma phenotype.

In conclusion, we claim that the scFv47 could serve as specific moiety for IL13Rα2-directed therapeutics, such as T and NK immune cells, fusion proteins, nano carriers, viruses, and other agents. As continuation of this study, we propose further development of Ad5FFscFv47 as an oncolytic agent alone or for targeted delivery of gene therapy such as suicide genes like HSV-tk and/or immunotherapy, such as checkpoint blockade with PD-1 or CTLA-4, directly to the tumor microenvironment^41^,^42^. This gene delivery and immunotherapy strategy has consistently demonstrated therapeutic efficacy in pre-clinical glioma models, and is currently being explored in human clinical trials.

Finally, the vast molecular heterogeneity of malignant gliomas has likely contributed to the lack of effective targeted therapies. Currently, there are several active and pending clinical trials designed for the personal therapy of patients with glioma. Eventually, with the development of an arsenal of targeted therapies, such as agents that specifically target and destroy IL13Rα2-expressing tumor cells and other tumor associated antigens such as EGFRvIII, personalized treatment protocols can be implemented in conjunction with traditional disease therapies like surgery and radiation to improve the outcome for patients with GBM.

## Materials and Methods

### Cell lines and reagents

Purified anti-c-*myc* antibody (clone 9E10) was obtained from the Frank W. Fitch Monoclonal Antibody Facility of the University of Chicago. Anti-M13–HRP antibody was obtained from Amersham Pharmacia Biotech (Uppsala, Sweden). Goat anti-mouse alkaline phosphatase conjugate was purchased from Sigma-Aldrich St. Louis, MO. Human embryonic kidney (HEK) 293, HEK 293- F28 stably expressing Ad5 wild type fibers, Chinese hamster ovary (CHO) cells, CHO-hCAR cells stably expressing human CAR, CHO-IL13Rα2^19^, U87MG and U251MG glioma cell lines were used. To generate IL13Rα2^+^ U251MG and IL13Rα2.KDU251MG, U251 cells were transduced with lenti viral particles encoding for control or IL13Rα2 shRNA (Sigma-Aldrich, St. Louis, MO). Transduced cells were selected with puromycin at 2 μg/ml and analyzed by flow cytometry for IL13Rα2 expression using our mAb IL13Rα2 (clone 47)^19^. Cells lines were cultured in DMEM media (Mediatech, Inc., Herndon, VA) supplemented with 10% heat inactivated FBS (Hyclone; Logan, UT) and penicillin-streptomycin (Mediatech, Inc., Herndon, VA). For formation of neurospheres, glioma cells were grown in neurobasal media (Life technologies, Eugene, OR) supplemented with EGF and bFGF at concentration of 20 ng/ml as well N10 and B27 supplements (Sigma-Aldrich, St. Louis, MO) for 7 days as described previously^43^. The patient-derived GBM43 and GBM39 glioma cells were obtained from Dr. Charles D. James from Northwestern University Feinberg School of Medicine. Cells were propagated via serial passaging in the flank of nude mice.

### Cloning the scFv fragment of mAb IL13Rα2 (clone 47)

The heavy and light chains of monoclonal antibody (clone 47) against human IL13Rα2^19^ were cloned using a set of gene specific primers (Supplementary Table 1) for immunogloblin heavy and light chains which were published previously^24^,^25^. The heavy and light chain cDNA were re-amplified to introduce *Nco*I and *Hin*dIII and *Mlu*I and *Not*I restriction sites, respectively for subsequent subcloning into phagemid vector pSEX 81 as previously described^44^. XL1 blue cells were transformed by electroporation with a library of scFv encoded by phage mid vector and phages were generated as previously described^25^. Selection of immune reactive phages was performed in three rounds using recombinant human IL13Rα2hFc fusion protein (rhIL13Rα2hFc) (R&D Systems) coated on 96-well plates. Human IgG served as a negative control. DNA of bacterial clones infected with positively selected phages was screened by PCR for the presence of proper size PCR products which consisted of full size scFv and plasmid sequences upstream and downstream of the scFv insert. Eight PCR products of approximately 1000 bp were purified for subsequent sequence analysis. The obtained scFv against human IL13Rα2 was designated as scFv47 elsewhere in the text reflecting the name of original clone of monoclonal antibody from which it was derived. In order to generate soluble protein, the cDNA encoding for scFv47 was re-cloned in a bacterial expression cassette as described previously^24^,^44^. XL-1 blue cells were utilized to generate soluble protein according to established protocol^24^.

### ELISA

Supernatant containing phages from all rounds of biopanning were analyzed for their binding specificity to rhIL13Rα2 protein using plate ELISA. Wells coated with human IgG were used as negative controls. Specifically, wells were blocked for 30 min with 2% non-fat dry milk and supernatants containing phages were applied at different dilutions to the wells. After a 30-min incubation with shaking and another 1.5 h without shaking, unbound phages were washed with PBS/0.05% Tween 20 and anti-M13 antibodies conjugated with peroxidase (Amersham Pharmacia Biotech, Uppsala, Sweden) (diluted 1/2000 in the 2% non-fat dry milk) were added. After intensive washing with PBS/0.05% Tween 20, the plates were developed with 1-step slow 3,3′5,5′-Tetramethyl-benzidine (TMB) substrate for ELISA and read at OD_450_ after the reaction was stopped with 3 N hydrochloric acid.

For analysis of soluble scFv47 for binding with rhIL13Rα2, clone of XL1 blue *E. coli* transformed with expression vector encoding for scFv47 gene tagged with c-myc and 6 His sequence was grown overnight in LB medium supplemented with 100 mM glucose and 100 μg/ml ampicillin (LB_GA_) as previously described^24^. Overnight culture was diluted 1/100 and grown in 50 ml of LB_GA_ media with shaking (250 rpm at 37 °C) until density OD_600_ = 0.8. After incubation, the bacterial culture was centrifuged at 1500*g* for 10 min. The pellet was re-suspended in 50 ml LB_GA_ media containing 0.4 M sucrose and 0.1 mM IPTG and grown for 20 h at room temperature (RT). The purified soluble scFv47 was obtained from the culture supernatant using Co resin per manufacturer’s recommendation (Thermo Scientific, Rockford, IL). Purified scFv47 was tested in plate ELISA and affinity studies as described below. 96 well plates coated with rhIL13Rα1 or rhIL13Rα2 protein at 1 μg/ml were blocked with 2% non-fat dry milk and scFv47 at various concentrations was applied for 2 h at RT. After washing, anti-*c-myc* monoclonal antibodies (clone 9E10) at 1 μg/ml were added for 1 h incubation, washed of unbound antibodies and subsequently developed with anti-mouse antibodies conjugated with alkaline phosphatase (Sigma Aldrich, St.Louis, MO). The reaction was then read in spectrophotometer at OD_405_.

### Competitive assay

Easy wash 96 well plates (Thermo Scientific, Rockford, IL) were coated with 100 μl of IL13Rα2hFc fusion protein at concentration of 1 ug/ml and stored overnight at + 4 °C. After washing with PBS/0.05% Tween 20, wells were blocked with 2% non-fat dry milk and incubated with either PBS or mouse IgG1 as negative controls or a panel of anti-IL13Rα2 mAbs recognizing non-overlapping epitopes with parental mAbIL13Rα2 (clone 47)^19^ at concentration of 2 μg/ml for 1 h. After washing of unbound antibodies, the supernatant containing phages after the third round of biopanning was applied to all wells for 2 h at RT. Bound phages were detected as described in the phage ELISA section above.

### Surface Plasmon Resonance and Kinetic Analysis

The affinity and rate of interaction between the scFv47 and target (rhIL13Rα2) were measured with a Biacore 3000 biosensor through surface plasmon resonance (SPR) as we previously described^19^. The estimation of kinetic parameters was performed by repetitive injections of a range of target concentrations over the immobilized rhIL13Rα2. Each data set, consisting of sensograms of increasing scFv47 concentrations over the same level of immobilized rhIL13Rα2, was analyzed using various kinetic models. The BIAevaluation v 4.1 software was used for data analysis. Affinity constants were estimated by curve fitting using a 1:1 binding model. Sensogram association and dissociation curves were fit locally or globally. The rate of complex formation during the sample injection is described by an equation of the following type: dR/dt = k_a_C (R_max_ − R) – k_d_R (for a 1:1 interaction) (R = SPR signal in RU; C = concentration of analyte; R_max_ = maximum analyte binding capacity in RU; dR/dt = rate of change of SPR signal). The early binding phase (300 sec) was used to determine the association constant (k_a_) between mAb and target. The dissociation phase (k_d_) was measured using the rate of decline in RU on introduction of free buffer at the end of target injections. Data were simultaneously fit by the software program (global fitting algorithm) and the dissociation constant (K_D_) of the complexes was determined as the ratio k_a_/k_d_. For quantitative analysis, 3 independent replicates were performed for each sample. Data are expressed as mean ± SEM.

### Adenoviral genetic modifications and virus production

In order to generate a fiber shuttle vector, the pKan 566 adenoviral genome (kindly gifted from Dr. Curiel at Washington University in Saint Louis), which contains the gene of fiber fibritin, was used. The cDNA of scFv47, was inserted at the C-terminal of fiber fibritin by using a standard molecular technique, resulting in pKan-scFv47FF^21^,^27^,^45^. With this fiber shuttle vector, recombinant HAd5 backbones containing an enhanced green fluorescence protein (eGFP) under the control of the CMV promoter in the E1 deleted region (replication incompetent Ad5scFv47FF-CMV-GFP) were generated. The recombinant virus were rescued in HEK293-F28 cells and then propagated in HEK293 cells. Viruses were purified by two rounds of CsCl gradient ultracentrifugation as previously described^27^.

### Flow Cytometry

In order to analyze expression of IL13Rα2 on the cell surface, U87MG and U251MG glioma cell lines were stained with IL13Rα2(clone 47) mAb at 2 μg/mL, followed by goat anti-mouse Alexa Fluor 647 (1:500). All staining procedures were performed on ice. Transduction of glioma cells with either control or Ad5FFscFv47 virus was assayed based on GFP expression. Samples were analyzed using the BD FACS Canto flow cytometer and FACS DiVa^TM^ software.

### PCR analysis

Viral DNA contained in Ad5-CMV-GFP and Ad5FFscFv47-CMV-GFP viral particles (10^9^) was used as the template for PCR amplification using a HAdV5-specific primer set: forward: 5′-CAGCTCCATCTCCTAACTGT-3′ and reverse: 5′-TTCTTGGGCAATGTATGAAA-3′ and scFv47-specific primer set: forward: 5′-CAGGTCCAACTGCAGCA-3′ and reverse: 5′-TTTGATTTCCAGCTTGGT-3′.

### Western blot

The supernatant of XL1-blue cells containing the scFv47 or purified adenoviral particles were diluted in Laemmli buffer, incubated either at room temperature (unboiled samples) or at 95 °C (boiled samples) for 10 mins and loaded to a 7.5% SDS-polyacrylamide gel (Bio-Rad, Hercules, CA). After electrophoretic separation, samples were transferred onto a PVDF membrane and detected with an anti-myc mAb (9E10) at 1 μg/ml for detection of soluble scFv47 or an anti-fiber tail mAb4D2 (1:3,000) (Thermo Scientific, Rockford, IL) followed by HRP tagged anti-mouse IgG secondary antibody (1:5,000) (Santa Cruz, CA, USA).

### Quantitative real-time real time-polymerase chain reaction (qRT-PCR) analysis

The expression of IL13Rα2 in U87MG cells growing as an adherent culture or as neurospheres was characterized by qRT-PCR. RNA was isolated from glioma cells using RNeasy plus kit (Qiagen, Boston, MA) and was reverse-transcribed using iScript cDNA conversion kit (Biorad, CA, USA). qRT-PCR reaction was carried out using SYBR green qPCR kit (Biorad, CA, USA) using following primers: GAPDH forward primer: 5′-GGTCGGAGTCAACGGATTTGG-3′; GAPDH reverse primer: 5′-CATGGGTGGAATCATATTGGAAC-3′; IL13Rα2 forward primer: 5′-TTGGGACCTATTCCAGCAAGGTGT-3′; IL13Rα2 reverse primer: 5′-CACTCCACTCACTCCAAATCCCGT-3′. For relative quantification of IL13Rα2 expression, all IL13Rα2 values were normalized to the glyceraldehyde 3 phosphate dehydrogenase (GAPDH) expression values. Data analysis was performed using the 2^−ΔΔCT^ method^46^.

### Viral Infectivity Analysis

Cells (3 × 10^5^) were plated in 24 well plates and incubated overnight. For monoclonal anti-IL13Rα2 mediated inhibition assay, 2 μg of anti-IL13Rα2 mAb (clone 47) was added to the cells and they were allowed to incubate for 1 h at 4 °C. Each virus sample was diluted to a multiplicity of infection of 300 viral particles (vp)/cell in 500 μl of infection media containing 2% FBS in DMEM. The cells were infected with Ad5FFscFv47-CMV-GFP for 2 h at 37 °C. Virus containing media was then replaced with the fresh media containing 2% FBS and cells were kept at 37 °C in atmospheric humidification containing 5% CO_2_ for 3 days, then flow cytometry analysis was performed as described above.

### Animal Experiments

Male athymic/nude mice were obtained from the Charles River Laboratory (Wilmington, MA) and were cared for in accordance with a study-specific animal protocol approved by the University of Chicago Institutional Animal Care and Use Committee. For tumor cell implantation, mice were anesthetized with a ketamine/xylazine mixture (115/17 mg/kg). A burr hole was made to allow for a stereotactic injection that was performed with a 10 μl Hamilton syringe (Hamilton, Reno, NV). The needle was mounted to a mouse-specific stereotactic, Harvard Apparatus (Holliston, MA) and was then inserted through the burr hole to an anatomical position of 3 mm in depth. Specifically, mice were implanted with intracranial glioma xenograft of IL13Rα2^+^ U251MG or with IL13Rα2.KDU251MG cells (5.0 × 10^5^cells). Ten days post-tumor implantation, mice were injected in the same coordinates as tumor cells with 10^9^ vp of Ad5FFscFv47-CMV-GFP in 5μl of PBS and sacrificed 3 days later.

### Confocal microscopy

Flash frozen brain tumor tissues were cut to a thickness of 10 μm. Tissue sections were fixed and stained for human nestin to visualize U251 glioma cells using mouse mAb (R&D systems, Minneapolis, MN). To analyze transduction of glioma cells with Ad5FFscFv47-CMV-GFP, transgene GFP expression was revealed using biotinylated anti-GFP antibody (Life technologies, Eugene, OR) and streptavidin-Alexa Fluor647 (Jackson ImmunoResearch, West Grove, PA). Cell nuclei were visualized using DAPI nuclear stain. Confocal microscopic images were captured with a 3i Marianas Yokogawa-type spinning disk confocal microscope with an Evolve EM-CCD camera (Photometrics, Tucson, AZ) running SlideBook v5.5 software (Intelligent Imaging Innovations, Denver, CO).

### Statistical Analysis

All statistical analyses were performed using Graphpad Prism 4 (GraphPad Software Inc., San Diego CA). Sample size for each group was ≥3 and numerical data were reported as Mean ± SEM. Student’s t test was used for comparisons between two groups, and ANOVA with Tukey’s post hoc test was used for comparisons between more than two groups. All reported *p* values were two-sided and were considered to be statistically significant at *p < 0.05, **p < 0.01, ***p < 0.001.

## Additional Information

**How to cite this article**: Kim, J. W. *et al.* A novel single-chain antibody redirects adenovirus to IL13Rα2-expressing brain tumors. *Sci. Rep.* **5**, 18133; doi: 10.1038/srep18133 (2015).

## Supplementary Material

Supplementary Information

## Figures and Tables

**Table 1:** Kinetics of scFv47 binding to the human recombinant IL13Rα2.

	k_a_ (1/Ms)	k_d_ (1/s)	K_D_ (M)	R_max_ (RU)
scFv47	3.08e3 ± 16	2.63e-6 ± 1.8e-8	0.9 · 10-9	496

The estimation of kinetic parameters was performed as described in the ‘Materials and Methods’. The dissociation constant (K_D_) of the complexes was determined as the ratio k_a_/k_d_. For quantitative analysis, 3 independent replicates were performed for each sample. Data are expressed as mean ± SEM. These data demonstrate that the affinity of scFv47 to rhIL13Rα2 is similar to parental mAb IL13Rα2 (clone 47) (1.39 · 10^−^9) as we have previously reported^19^.
