# Supplementary material for: A novel single-chain antibody redirects adenovirus to IL13Rα2-expressing brain tumors
Source: Sci Rep. 2015 Dec 14;5:18133. doi: 10.1038/srep18133 (PMC4677343; doi:10.1038/srep18133)
Supplement: Supplementary Information [file srep18133-s1.pdf]

Supplementary Information

A novel single-chain antibody redirects adenovirus to IL13Rα2-expressing brain tumors.

Julius W. Kim, Jacob S. Young, Elena Solomaha, Deepak Kanojia, Maciej S. Lesniak, Irina V. Balyasnikova.

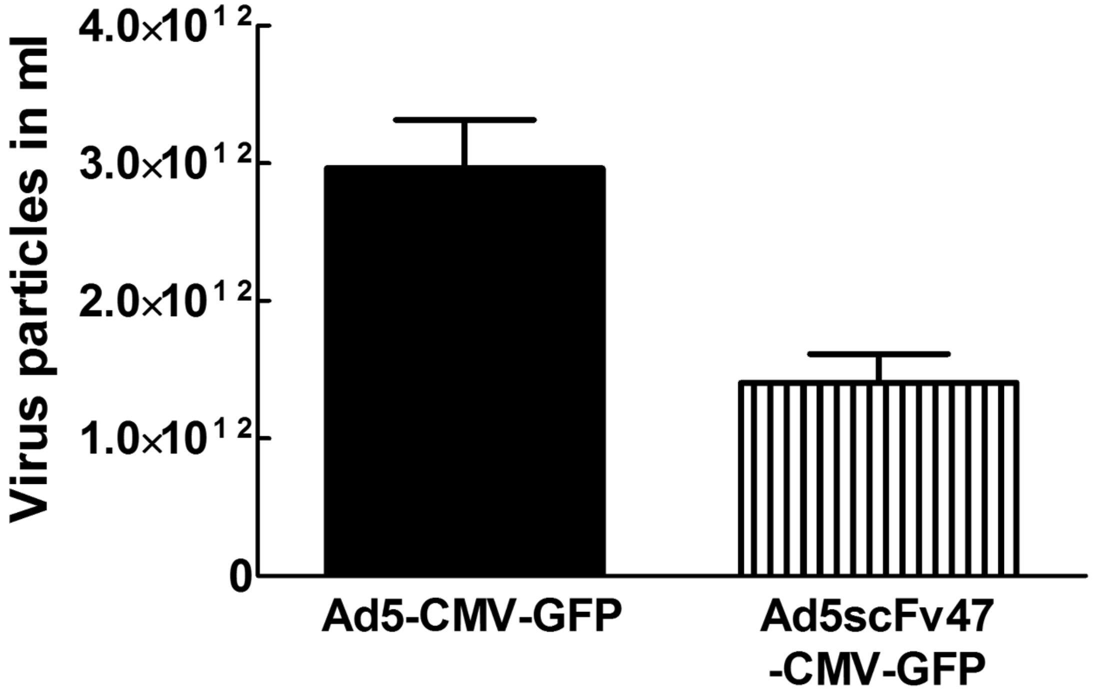

Supplementary Figure 1. The efficiency of Ad5FFscFv47-CMV-GFP viral production. Both Ad5-CMV-GFP and Ad5FFscFv47-CMV-GFP were propagated, purified and titrated as described in the Material and Method section. Each data point is an average of 3 independent replicates. Mean ± SEM is plotted.

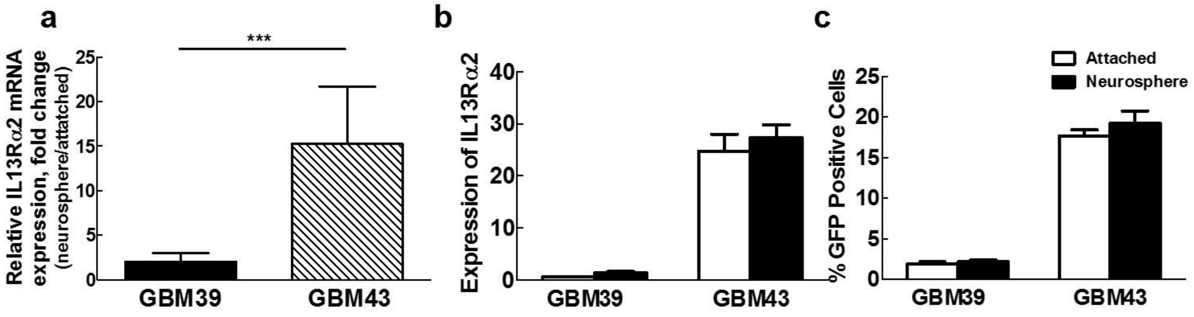

Supplementary Figure 2. Infection of primary GBM cells by Ad5FFscFv47-CMV-GFP virus. A. Relative IL13Rα2 mRNA expression in patient derived primary GBM39 and GBM43 cells grown as adherent or neurosphere cultures was analyzed by qRT-PCR. The level of IL13Rα2 mRNA expression was normalized to GAPDH mRNA expression. **B.** Comparison of IL13Rα2 expression on the surface of the primary GBM cells grown as an adherent culture or as neurospheres. **C.** Ad5FFscFv47-CMV-GFP infectivity of the primary GBM cells grown as adherent or neurospheres cultures determined by flow cytometry analysis for GFP-positive cells. Each data point is an average of 3 independent replicates. Mean ± SEM is plotted. *** $P<0.001$.

Supplementary Table 1. Primer sets used for amplification of variable regions of antibody [a].

	**Heavy chain**	**Light kappa chain**
Forward 1	5'-GATGTGAAGCTTCAGGAGTC-3'	5'-GATGTTTTGATGACCCAAACT-3'
Forward 2	5'-CAGGTGCAGCTGAAGGAGTC-3'	5'-GATATTGTGATGACGCAGGCT-3'
Forward 3	5'-CAGGTGCAGCTGAAGCAGTC-3'	5'-GATATTGTGATAACCCAG-3'
Forward 4	5'-CAGGTTACTCTGAAAGAGTC-3'	5'-GACATTGTGCTGACCCAATCT-3'
Forward 5	5'-GAGGTCCAGCTGCAACAATCT-3'	5'-GACATTGTGATGACCCAGTCT-3'
Forward 6	5'-GAGGTCCAGCTGCAGCAGTC-3'	5'-GATATTGTGCTAACTCAGTCT-3'
Forward 7	5'-CAGGTCCAACTGCAGCAGCCT-3'	5'-GATATCCAGATGACACAGACT-3'
Forward 8	5'-GAGGTGAAGCTGGTGGAGTC-3'	5'-GACATCCAGCTGACTCAGTCT-3'
Forward 9	5'-GAGGTGAAGCTGGTGGAATC-3'	5'-CAAATTGTTCTCACCCAGTCT-3'
Forward 10	5'-GATGTGAACTTGGAAGTGTC-3'	5'-GACATTCTGATGACCCAGTCT-3'
Forward 11	5'-GAGGTGCAGCTGGAGGAGTC-3'	n/a
Reverse	5'-GGCCAGTGGATAGTCAGATGGGGGTGTCGTTTTGGC-3'	5'-GGATACAGTTGGTGCAGCATC-3'

[a] Sequences of primer sets were previously published[25]
